# Experiments with the Electro-Deposit Plate

**Published:** 1890-04

**Authors:** 


### EDITORIAL.

WE have recently been making some experiments with the electro deposit plate for mounting artificial dentures. These plates are made of pure gold deposited by the electro current, directly upon the models of the gums to which the plate is to be adapted, and so far as our experiments have extended they are eminently satisfactory. If the impression has been correctly obtained the plate, of necessity, is a perfect fit, equal to, if not better, than those made by any other method. They seem to have strength equal to that of the wrought plate. The teeth are attached by rubber and when properly mounted the result is beautiful and efficient work. Some question has been raised as to the durability of these plates. Their strength seems to be entirely sufficient, and so far as we can see, there appears no reason why they should not be as permanent as any gold plate. This question time and experience can only reveal; of course, it is possible that a weak point may ultimately appear, but that has not yet been detected.

We are so well impressed with these plates that we have no hesitancy in recommending every one to make a trial of them, for we are fully convinced that all will recognize upon trial the good points which we have mentioned.

